# Sepiapterin reductase promotes hepatocellular carcinoma progression via FoxO3a/Bim signaling in a nonenzymatic manner

**DOI:** 10.1038/s41419-020-2471-7

**Published:** 2020-04-20

**Authors:** Yao Wu, Hongzhi Du, Meixiao Zhan, Hongxv Wang, Peng Chen, Danyu Du, Xinyi Liu, Xingxv Huang, Pengcheng Ma, Dezheng Peng, Li Sun, Shengtao Yuan, Jian Ding, Ligong Lu, Jingwei Jiang

**Affiliations:** 1 Jiangsu Key Laboratory of Drug Screening, China Pharmaceutical University, Nanjing, China; 2 School of Pharmacy, Hubei University of Chinese Medicine, No.16, Huangjiahu Road West, Wuhan, China; 3 Zhuhai Interventional Medical Center, Zhuhai Precision Medical Center, Zhuhai People’s Hospital, Zhuhai Hospital Affiliated with Jinan University, Zhuhai, China; 4 Department of Neurosurgery, The Second Affiliated Hospital of Nanchang University, Nanchang, China; 5 School of Life Science and Technology, ShanghaiTech University, 100 Haike Road, Pudong New Area, Shanghai, China; 6 Institute of Dermatology, Chinese Academy of Medical Science, Peking Union Medical College, 12 Jiangwangmiao Street, Nanjing, China; 7 State Key Laboratory of Drug Research, Shanghai Institute of Materia Medica, Chinese Academy of Sciences, Shanghai, China

**Keywords:** Targeted therapies, Oncogenes

## Abstract

Sepiapterin reductase plays an enzymatic role in the biosynthesis of tetrahydrobiopterin, which is reported in limited studies to regulate the progression of several tumors. However, the role of sepiapterin reductase in hepatocellular carcinoma remains largely unknown. Here, we found that sepiapterin reductase was frequently highly expressed in human hepatocellular carcinoma, which was significantly associated with higher T stage, higher tumor node metastasis stage, and even shorter survival of hepatocellular carcinoma patients. Furthermore, cell and animal experiments showed that sepiapterin reductase depletion inhibited cancer cell proliferation and promoted cancer cell apoptosis. Importantly, the results suggested that sepiapterin reductase enzymatic activity was not necessary for the progression of hepatocellular carcinoma, based on the comparison between SMMC-7721 and SMMC-7721 containing sepiapterin reductase mutant. Moreover, we showed that sepiapterin reductase regulated the development of hepatocellular carcinoma via the FoxO3a/Bim-signaling pathway. Collectively, our study suggests that sepiapterin reductase controls hepatocellular carcinoma progression via FoxO3a/Bim signaling in a nonenzymatic manner, which provides a potential prognostic factor and therapeutic strategy for hepatocellular carcinoma.

## Introduction

Metabolic reprogramming is considered to be a core hallmark of tumors, and cancer cells rely on metabolic changes to support their rapid growth^1,2^. Therefore, characterization of reprogrammed metabolism provides opportunities to suppress cancer development^3,4^. According to previous reports, tetrahydrobiopterin (BH_4_; 6R-L-erythro-5,6,7,8-tetrahydrobiopterin), which is involved in the process of phenylalanine catabolism^5^, ether lipid metabolism^6^, and immunometabolism^7^, acts as a regulator in cell metabolism. In addition, it has been reported that BH_4_ plays a key role in the growth, angiogenesis, and migration of tumors^8–10^. Sepiapterin reductase (SPR), which is an important regulator in the biosynthesis of BH_4_^11^, has been demonstrated to be significantly correlated with survival of human neuroblastoma (NB)^12^. Although NB cells treated with SPR small interfering RNA (siRNA) or an enzymatic inhibitor showed a significant decrease in growth, the underlying mechanism has not been fully elucidated^12–14^, and the biological functions and molecular mechanisms of SPR in most types of tumors remain unknown. Among them, hepatocellular carcinoma (HCC) is a malignancy with poor prognosis, and is one of the leading causes of cancer death in China. Based on these observations, we aimed to determine whether SPR could be a diagnostic biomarker for HCC.

Because of its critical role, many studies of inhibitors have focused on the enzymatic activity of SPR, such as sulfhydryl reagents developed in the last few decades^10,15–18^. The effects of SPR inhibitors on cancer progression depend on the type of tumor. For instance, sulfasalazine, an inhibitor of SPR enzymatic activity, suppressed the proliferation of NB cells and exerted synergistic anti-proliferative effects in combination with α-difluoromethylornitine^12^. However, the depletion of BH_4_ using a SPR inhibitor had no effects on MOLT-4 and MCF-7 cancer cell growth^19^. The reason for such contradictory results in different cancers might be that the enzymatic function of SPR could be bypassed by aldose reductase and carbonyl reductase in the liver and other peripheral tissues, rather than in the central nervous system^20^. In addition, SPR inhibitors use has the potential risk of inducing hyperphenylalaninemia and several neurological diseases via suppression of BH_4_ biosynthesis^21,22^. Taken together, it is necessary to identify a new mechanism of SPR to provide a potential target for tumor therapy.

Considerable evidence suggests that many enzymes are involved in tumor development independent of their catalytic activities. For example, the M2 isoform of pyruvate kinase, aside from its pyruvate kinase activity, promotes cyclin D1 expression through binding to β-catenin^23^. In addition to its glycolytic enzymatic activity, phosphoglycerate mutase 1 also directly interacts with α-smooth muscle actin and modulates cancer cell migration^24^. Increasing evidence has also indicated that the interaction between lysine-specific histone demethylase 1 (LSD1) and the transcription factor, GFI1B, instead of the demethylase activity of LSD1, is necessary for acute myeloid leukemia survival^25,26^. These results emphasize that the catalytically independent activities of enzymes play important roles in cancer progression and deserve careful characterization.

In the present study, different approaches were used to reveal the role of SPR and explore the nonenzymatic function of SPR in HCC progression. Clinical data showed that SPR levels were higher in HCC tissues than the adjacent nontumor liver tissue, indicating that SPR was a possible tumor promotor in HCC. In addition, cell biology experiments and a nude mice xenograft model showed that SPR depletion suppressed HCC cell proliferation and promoted cell apoptosis. Furthermore, SPR enzymatic inhibitors and genome-editing technology were used to show that SPR enzymatic activity was not necessary for the progression of HCC. We also found that Bim expression was upregulated by SPR knockdown via facilitating the nuclear translocation of forkhead box protein O3 (FoxO3a). In brief, our study characterized the clinical implications, functional significance, and nonenzymatic molecular mechanism of SPR in HCC, which could provide a novel clinical prognostic indicator and therapeutic target for this type of tumor.

## Material and methods

### Cell culture

Human liver cancer cell lines BEL-7402 and SMMC-7721 were obtained from BeNa Culture Collection (Beijing, China). These cells were cultured according to the supplier’s instructions. Furthermore, these cells were authenticated by short tandem repeat and tested for mycoplasma contamination (BeNa Culture Collection, Beijing, China) in recent years

### Tissue microarray and immunohistochemistry

Human liver cancer tissue microarray (HLiv-HCC197sur-01) was provided by National Human Genetic Resources Sharing Service Platform (Shanghai, China). In the microarray, 97 liver cancer tissues and 89 adjacent liver tissues were conducted in formalin-fixed paraffin-embedded sections. Tissue specimens used in this study were reviewed and approved by the Committees for Ethical Review of Research at Taizhou Hospital of Zhejiang Province (Zhejiang, China) and informed consent was obtained from all patients. IHC staining with SPR-specific antibody was performed using DakoCytomation EnVision^+^ System-HRP (DAB; DakoCytomation California, Inc., Carpinteria, CA) detection kit according to the manufacturer’s instructions. The stained slides were observed under a microscope and images were acquired. The immunoreactivity of each sample was blindly quantified by a histochemical score. Briefly, the score was calculated as the sum of the intensity of staining (0 for negative, 1 for weakly positive, 2 for moderately positive, and 3 for strongly positive) multiplied by the percentage of cells stained (0 for 0%, 1 for 1–25%, 2 for 26–50%, 3 for 51–75%, and 4 for 76–100%). The intensity and percentage were obtained by two analysts who did not know the information of the samples.

### siRNA and antibodies

SPR siRNA (siSPR#1 and siSPR#2), siRNA targeting Bim (siBim) or nontargeting siRNA (siNC) were purchased from Shanghai GenePharma Co., Ltd (Shanghai, China). The sequences of siSPR#1 and siSPR#2 are 5′-GACUGCUGCUUAUCAACAATT-3′ and 5′-GCUGCUCGUGAUAUGCUGUTT-3′. Sequence of BIM is 5′-GCCACAAGGUAAUCCUGAATT-3′. Sequence of NC is 5′-UUCUCCGAACGUGUCACGUTT-3′. Anti-SPR (ab157194), anti-FoxO3a (ab12162), anti-phospho-FoxO3a (ab154786), and anti-SP1 (ab124804) were purchased from Abcam (Cambridge, MA, USA). Anti-PARP (#6704), anti-Caspase 3 (#9665), anti-Bim (#2933), anti-phospho-Bim (#4585), anti-Bax (#2774), anti-phospho-Erk (#4370), anti-VDAC (#4661), anti-β-Tubulin (#2146), and anti-Lamin B1 (#12586) were obtained from Cell Signaling Technology (Danvers, MA, USA). Anti-Cytochrome C (10993-1-AP) was purchased from Proteintech. Anti-β-Actin (AC026) and anti-GAPDH (AC001) were obtained from Abclonal Technology.

### Western blot, RT-PCR, and immunofluorescence assay

After treatment for 48 h, cellular proteins were extracted and western blot was performed. The total cellular RNA was isolated with the TRIzol Reagent (Vazyme Biotech, Nanjing, China) and reverse transcribed with the HiScript QRT SuperMix for quantitative PCR (qPCR; Vazyme Biotech). The mRNA levels were measured with the SYBR Green master mix (Vazyme Biotech). The immunofluorescence assay was performed according to previous reports^27^.

### In vitro functional assays

A real-time cell analyzer (RTCA) (xCelligence RTCA SP; Roche Diagnostics GmbH, Penzberg, Germany) was used to assess cell proliferation according to the manufacturer’s instructions. Briefly, 5 × 10^3^ cells were seeded onto the RTCA’s E-96 plate. After 30 min of equilibrating, the plate was transport to RTCA and electrode resistance was recorded.Twenty-four hours later, cells were treated with siSPR and cultured for 8 h. After that, the media were changed and cell growth was monitored continuously up to another 72 h. MTT and colony formation assays were performed as previously described^28^. Apoptosis, cell cycle, and mitochondrial membrane potential were measured using annexin V-FITC apoptosis detection kit (Vazyme Biotech), Cell cycle and apoptosis analysis kit (Beyotime Biotechnology, Shanghai, China) or mitochondrial membrane potential assay kit (Beyotime Biotechnology) according to the manufacturer’s instructions.

### Plasmids

The pLVX-Puro-SPR expression vector was obtained from Public Protein/Plasmid Library. To produce recombinant SPR or SPR^D257G^ with histidine tags, the SPR gene or SPR^D257G^ gene was cloned into a pET-28b (GenScript, Piscataway, Nanjing, China) expression plasmid. For the construction of sgRNAs, oligos were synthesized, annealed, and cloned into the BsaI site of pGL3-U6-sgRNA-PGK-puromycin (Addgene, 51133) vector. pCMV-ABE and pST1374-N-NLS-flag-linker-Cas9 plasmid were kindly provided by Pro. Xingxv Huang (ShanghaiTech University, Shanghai, China).

### Expression and purification of recombinant SPR or SPR^D257G^

*E. coli* strain BL21 (DE3) obtained from Tiangen Biotech Co., Ltd (Beijing, China) was transformed with the plasmid and cultured in selective antibiotic LB agar plate. After 16 h, a single colony was picked and cultured in 10 mL LB medium containing 50 μg/mL kanamycin with vigorous shaking at 37 °C for 10 h. Then the 10 mL cultures were added to 250 mL media and cultured for 2 h. Next, protein expression was induced by adding IPTG to a final concentration of 0.5 mM. The cells were left to grow overnight at 16 °C and then harvested by centrifugation. The extraction and purification of protein were performed using Ni-NTA Fast Start Kit (QIAGEN, Duesseldorf, Germany). Then the purified protein was concentrated by Centrifugal Filter Devices (Merck Millipore, Billerica, MA, USA) and mixed with glycerol to a final concentration of 20%, and stored at −80 °C until used.

### Enzyme assay

The assay was performed in 50 mM potassium phosphate pH 6.5. Firstly, 20 μL of water or relevant inhibitor in water was added in a transparent 384-well plate (Corning Incorporated, Corning, NY, USA). Then 45 μL of enzyme mix, containing 100 μg/mL BSA, 200 μM NADPH, 2.5 ng/μL SPR, and 50 mM potassium phosphate pH 6.5, was added. Next, 15 μL of 100 μM l-sepiapterin (Santa Cruz Biotechnology) in potassium phosphate was added. Absorbance at 420 nm was detected after 1 h of reaction at 37 °C.

### SPR editing and knockout of FoxO3a by CRISPR/Cas9 system

Adenine base editor (ABE) and SPR-targeting sgRNA, whose sequence was 5′-GTGGACTTCTATGACAAATAAGC-3′, were transfected into cells and selected by puromycin (1 μg/mL). The selected cells were seeded into 96-well plates for single colony growth. Then, the mutation of SPR in picked clones was validated by Sanger sequencing. To construct FoxO3a-depleted cells, Streptococcus pyogenes Cas9 (SpCas9) and FoxO3a targeting sgRNA (5′-GCGTTGCGTGCCCTACTTCA-3′) were infected into cells. Puromycin (1 μg/mL) and blasticidin (10 μg/mL) were added to cells after 48 h of transfection. Seventy-two hours later, the selected cells were cultured in normal media. Western Blot and qPCR were conducted to confirm the knockdown of FoxO3a in picked cells.

### Transcriptome sequencing

The global gene expression profiles of wild type and SPR knockdown SMMC-7721 cells were examined by RNA sequencing (RNA-seq). Gene expressions were analyzed with The Subread package (http://subread.sourceforge.net/) and the differential expression analysis was performed using edgeR (http://www.bioconductor.org/packages/release/bioc/html/edgeR.html). Pathway analyses were carried out with gene set enrichment analysis (GSEA, http://software.broadinstitute.org/gsea/index.jsp).

### Nude mice xenograft model

Four- to six-week-old male Balb/c mice (MARC, Nanjing University, Nanjing, China) were housed under specific pathogen-free conditions and cared for in accordance with protocols approved by the Experimental Animal Care Commission in China Pharmaceutical University. SMMC-7721 cells (1.0 × 10^6^) were injected subcutaneously into the right flank of mice. When the volume of tumors reached about 100 mm^3^, mice were randomly allocated (six mice per group) and treated with multipoint intratumoral injection of siRNA (10 μg per tumor) complexed with in vivo-jetPEI transfection reagent (Polyplus-transfection Inc., New York, NY, USA) every other day. Tumor volumes were monitored throughout the experiment. Mice were sacrificed after 2 weeks of treatment, tumors were removed, photographed, and processed for immunohistochemical and western blot analysis.

### Statistical analysis

Statistical analyses were performed using SPSS 19.0 (SPSS, Chicago, IL, USA) and Prism 5.0 (GraphPad Software, La Jolla, CA, USA) software. Data are presented as the mean ± standard deviation of at least three independent experiments. Quantitative data were evaluated by the Student's *t*-test. Categorical data were compared using the *χ*^2^ test or Fisher exact test. Kaplan–Meier and log-rank methods were used to estimate the survival disparity between different subgroups. Cox proportional hazards regression was used for multivariate survival analysis. A two-tailed *p* values of < 0.05 were considered statistically significant.

## Results

### SPR is overexpressed in HCC and is correlated with poor prognosis

**Fig. 1:**
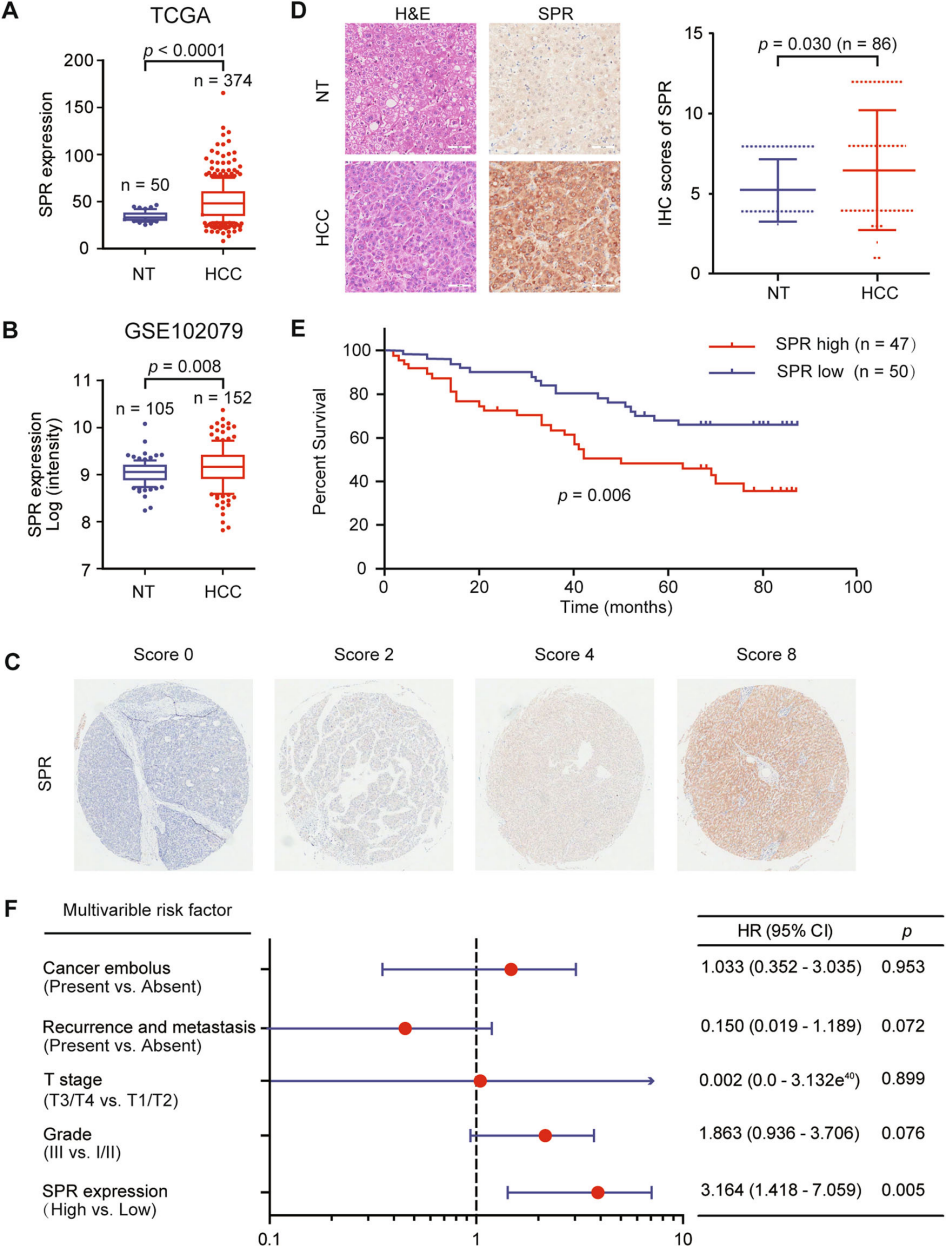
High SPR expression correlates with poor prognoses in HCC patients. Upregulated expression of SPR in HCC tissue compared to paired nontumor tissues in **a** TCGA (*n* = 374) and **b** GSE102079 (*n* = 152) datasets. **c** and **d** The expression level of SPR was consistently analyzed in human liver and HCC specimens. Scale bar: 50 μm. **e** Kaplan–Meier survival analysis of the prognostic value of SPR based on the expression data of HCC patients (*n* = 97). **f** Multivariate Cox regression analysis of clinicopathological factors for overall survival in the HCC patients (*n* = 97). CI confidence interval, HR hazard ratio, NT nontumor tissues.

To identify the role of SPR in HCC, we conducted bioinformatic analyses based on different datasets. According to The Cancer Genome Atlas (TCGA; Fig. 1a; https://portal.gdc.cancer.gov/) and Gene Expression Omnibus (Fig. 1b; GSE102079, https://www.ncbi.nlm.nih.gov/gds/) datasets, SPR mRNA levels were markedly upregulated in HCC tissues compared to their counterparts. Furthermore, we analyzed SPR expression levels by immunohistochemistry on a tissue microarray containing 97 liver cancer tissues and 89 adjacent liver tissues. In this process, SPR immunoreactivity was graded as negative (score 0), low (score 1–4), and high (score > 4) (Fig. 1c). Among the 86 matched cases examined, positive SPR expressions were detected in 82 of 86 (95.3%) HCC tissues. In the 82 positive HCC samples, 42 (51.2%) specimens showed strong staining of SPR. Moreover, the average SPR protein level was significantly higher in tumor tissues relative to the corresponding adjacent nontumor liver tissues, indicating that SPR was frequently overexpressed in HCC (Fig. 1d).

Correlative analysis of SPR protein levels with clinicopathological features in 97 HCC patients suggested a significant association between SPR expression and age (*p* = 0.02), T stage (*p* = 0.019), tumor node metastasis stage (*p* = 0.019), and sex (*p* = 0.029; Table 1). Additionally, Kaplan–Meier analysis indicated that patients with high SPR expression in tissues had significantly shorter overall survival percentages (*p* = 0.006; Fig. 1e). Furthermore, univariate Cox regression analysis revealed that SPR expression, grade, T stage, recurrence, distant metastasis, and cancer embolus were significantly associated with overall survival. Multivariate Cox regression analysis also showed that SPR expression was an independent predictor of the overall survival of HCC patients (Fig. 1f). Taken together, SPR is frequently highly expressed in HCC and plays a potential tumor-promoting role in this cancer.

**Table 1:** Correlation between SPR expression and clinicopathological characteristics.

Variables		SPR expression		Total	*χ*^2^	*p* value
		High	low			
Age (year)					5.445	**0.020**
	≤58	18	31	49		
	>58	29	19	48		
T stage					5.461	**0.019**
	T1/T2	24	37	61		
	T3/T4	23	13	36		
TNM stage					5.461	**0.019**
	Ι/II	24	37	61		
	III/IV	23	13	36		
Sex					4.784	**0.029**
	Female	3	11	14		
	Male	44	39	83		
Cancer embolus					0.471	0.492
	No	28	33	61		
	Yes	13	11	24		
	Null	12				
Cirrhosis					0.008	0.927
	No	10	11	21		
	Yes	33	38	71		
	Null	5				
Grade					0.849	0.357
	I/II	36	35	71		
	III	10	15	25		
	Null			1		
CD34						0.493^a^
	Negative	0	2	2		
	Positive	31	31	62		
	Null			33		
CK19					0.042	0.838
	Negative	32	35	67		
	Positive	15	15	30		
Recurrence or metastasis					0.535	0.464
	No	40	45	85		
	Yes	7	5	12		

*TNM* tumor node metastasis.

^a^Fisher exact probability test.

Statistically significant values are shown in bold.

### SPR regulates the proliferation and apoptosis of HCC cells

**Fig. 2:**
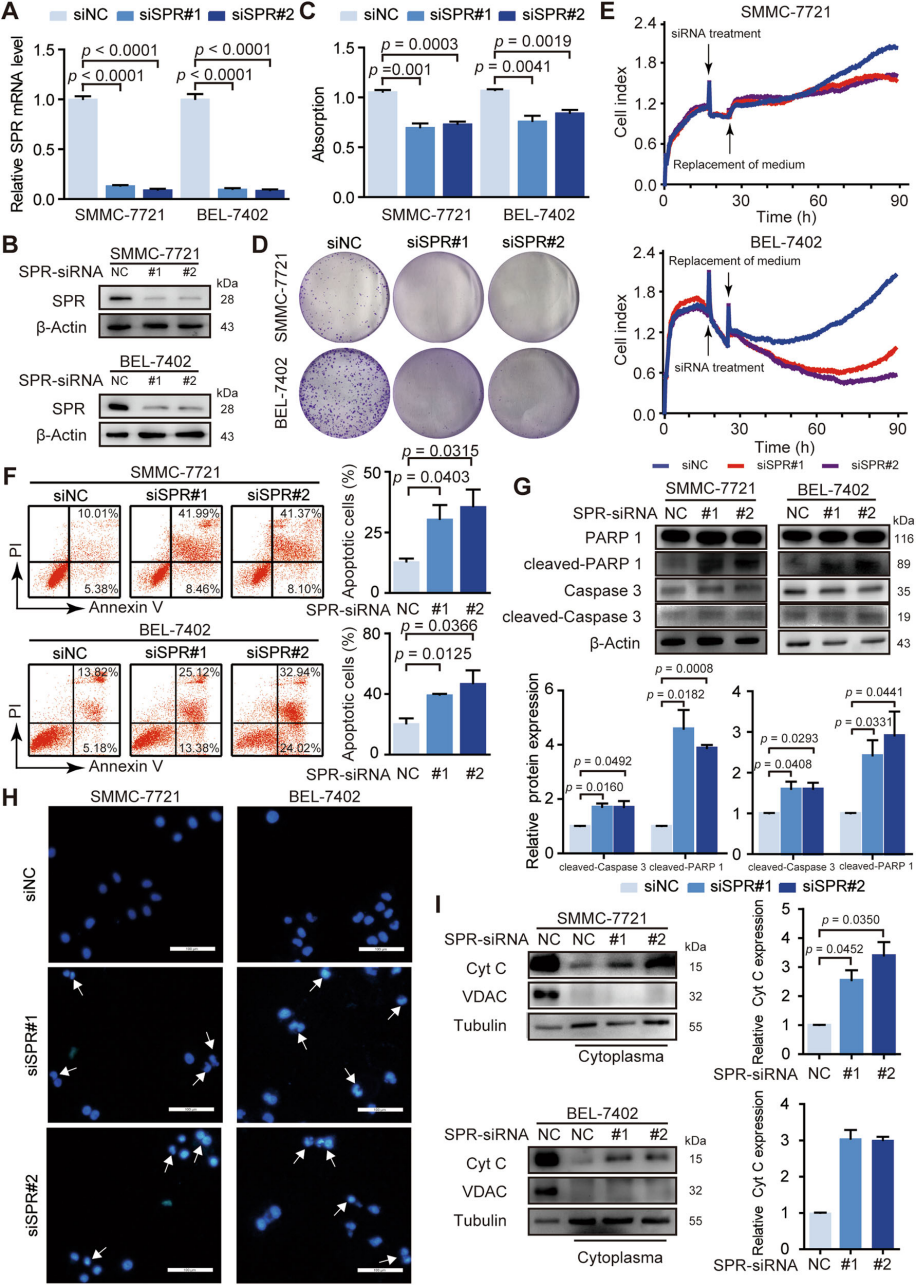
SPR acts as an oncogene in HCC progression. The knockdown efficiency of siRNAs was determined by **a** RT-PCR and **b** western blotting. **c** MTT assays were performed to determine the proliferation of SMMC-7721 and BEL-7402 cells transfected with siRNAs. **d** The effects of silencing SPR on cell proliferation of SMMC-7721 and BEL-7402 cells were detected by colony formation assays. **e** RTCA assays were performed to evaluate the effect of SPR knockdown on HCC cell proliferation. **f** Cell apoptosis was measured by flow cytometry using annexin V/PI staining in cells after siSPR treatment. **g** Apoptosis in different groups was analyzed by PARP cleavage and Caspase 3 cleavage analysis using western blots. **h** Hoechst staining of the cellular nuclei was used to identify apoptosis in HCC cells. Scale bar: 100 μm. **i** The level of cytochrome C (Cyt C) in the cytoplasm was detected in SMMC-7721 and BEL-7402 cells transfected with siSPR. Data are represented as the mean ± SD of three independent experiments. The *p*-values < 0.05 were considered statistically significant for all tests.

Based on these observations, we characterized the biological functions of SPR in HCC. The siRNA-mediated loss of function study in HCC cells showed that transfection with two siRNAs (siSPR#1 and siSPR#2) resulted in more than 80% decrease in the *SPR* gene and protein expression in SMMC-7721 and BEL-7402 cells (Fig. 2a, b). MTT assays revealed that knockdown of SPR inhibited proliferation of HCC cells (Fig. 2c). Furthermore, colony formation assays indicated that decreased SPR expression significantly suppressed the proliferation of HCC cells (Fig. 2d). Consistent with these results, proliferation curves detected by the RTCA confirmed that HCC cell growth was impaired by SPR knockdown (Fig. 2e). Moreover, HCC cells that stably overexpressed SPR (Fig. S1A,B) exhibited remarkably increased colony formation relative to cells transfected with empty vector (Fig. S1C). The proliferation curves showed increased proliferation in SPR-overexpressing liver cancer cells (Fig. S1D). Collectively, these results suggest that SPR promotes the proliferation of HCC cells.

We also determined the effects of SPR on HCC cell apoptosis. Annexin V/propidium iodide (PI) assays showed a dramatic increase in the apoptosis percentage in SPR-depleted cells compared with their respective controls (Fig. 2f). SPR depletion-induced apoptosis was further shown by enhanced PARP cleavage and Caspase 3 cleavage (Fig. 2g). Consistent with these results, HCC cells transfected with siRNA showed reduced nuclear size, strong fluorescent spots and pyknotic nuclei after Hoechst staining (Fig. 2h). Additionally, a decrease in mitochondrial membrane potential of HCC cells was induced by SPR silencing (Fig. S1E). Moreover, more cytochrome c, which is released from the mitochondria to the cytosol during the early stage of mitochondria-dependent apoptosis, was detected in the cytosolic fraction of HCC cells treated with siRNA compared with control cells (Fig. 2i). However, SPR knockdown did not affect the cell cycle (Fig. S1F). Overall, SPR is essential for HCC cell proliferation and mitochondria-dependent apoptosis.

### SPR loss promotes HCC cell apoptosis independently of its enzymatic activity

**Fig. 3:**
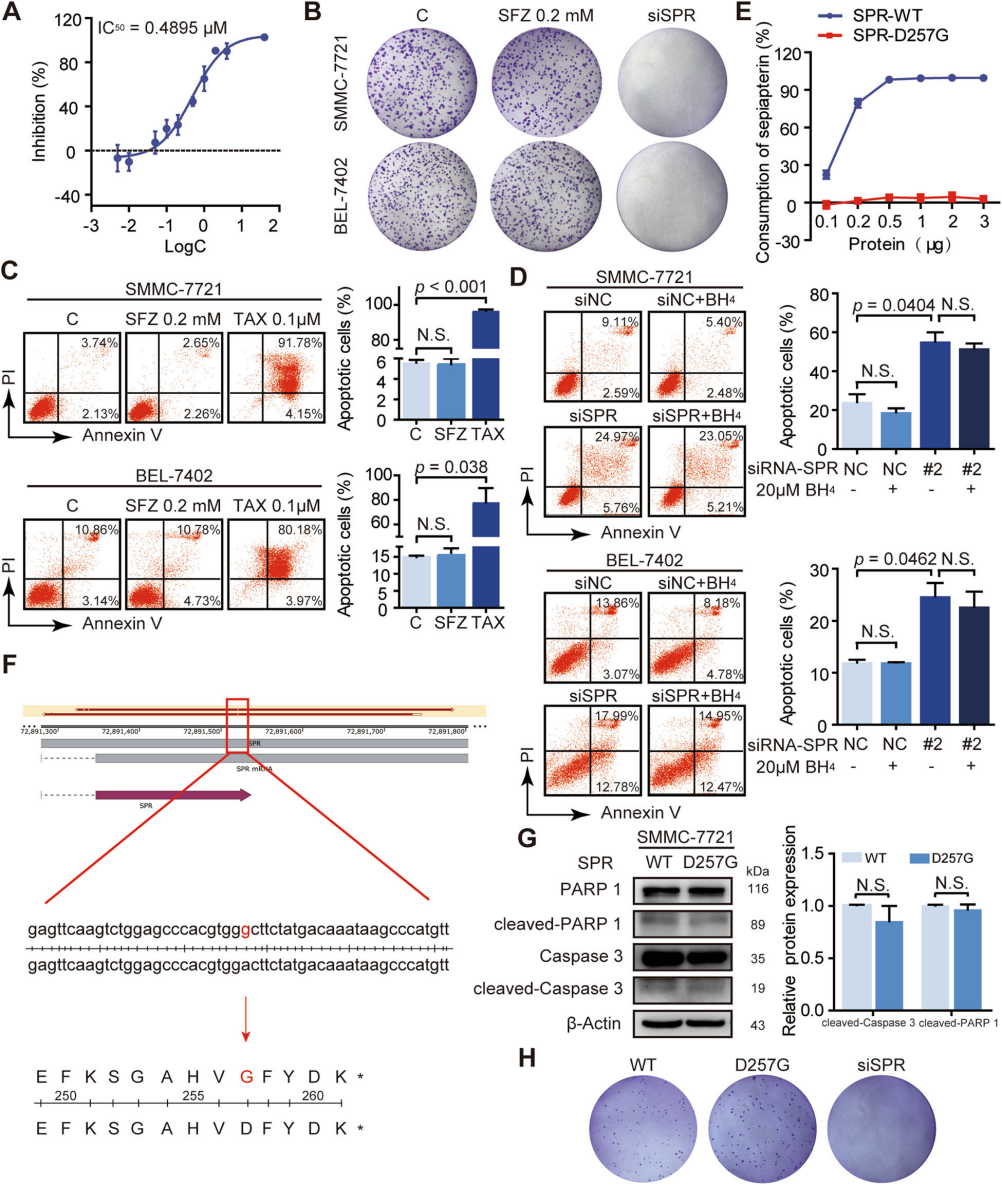
SPR promotes HCC progression independent of its enzymatic activity. **a** The inhibitory activities of sulfathiazole (SFZ) against human SPR were measured in a spectrophotometric assay. **b** The effects of SFZ on cell viability of SMMC-7721 and BEL-7402 cells were detected using colony formation assay. **c** Cell apoptosis was detected by flow cytometry after treatment with SFZ at the indicated concentrations for 96 h in SMMC-7721 and BEL-7402 cells. **d** The effect of BH_4_ on cell apoptosis of SMMC-7721 and BEL-7402 cells transfected with siSPR. **e** The enzymatic activity of SPR in the wild type (WT) and indicated SPR mutant at different protein amount titrations in the biochemistry enzymatic assay. **f** The point mutant of SPR in SMMC-7721 was confirmed by Sanger sequencing. **g** Apoptosis in SMMC-7721 and SMMC-7721/SPR D257G cells was analyzed by detecting the PARP cleavage and Caspase 3 cleavage level. **h** The colony formation assay of SMMC-7721 with the indicated mutant. Data are represented as the mean ± SD of three independent experiments. The *p*-values < 0.05 were considered statistically significant for all tests. TAX paclitaxel.

Because the enzymatic activity of SPR, which modulates BH_4_ biosynthesis, has been reported to influence NB cell growth^13,14^, the effects of SPR catalytic activity on HCC cell proliferation and apoptosis were determined. Treatment with two SPR enzymatic inhibitors, sulfathiazole and sulfapyridine, failed to affect cell proliferation and apoptosis, despite diminished SPR enzymatic activity (Figs. 3a–c; S2A–C). Similarly, SPR depletion-induced apoptosis of HCC cells was not reversed by supplementation of BH_4_ (Fig. 3d). These results confirm that SPR can function in HCC independently of its enzymatic activity.

For further confirmation, we constructed and purified a catalytically inactive SPR mutant (D257G) based on the report by Yang et al.^29^. The enzymatic activities of SPR and its mutant were measured by an enzyme-coupled assay with purified recombinant proteins, using sepiapterin conversion as the readout. The D257G mutant of SPR completely lost catalytic activity compared with the wild-type enzyme (Fig. 3e). The structure of the D257G mutant was also analyzed by homology modeling (Fig. S2D; https://swissmodel.expasy.org/), which showed that there was no significant difference in structure between the SPR mutant (D257G) and SPR (GMQE = 0.99; QMEAN = 1.55). Furthermore, HCC cells with a SPR point mutation (HCC/SPR^D257G^) were constructed by transfecting SMMC-7721 cells with plasmids encoding ABE and the SPR^D257G^ sgRNA. The selected cell clones were validated by Sanger sequencing (Figs. 3f; S2E). Moreover, western blot assays indicated that loss of SPR enzymatic activity did not affect apoptosis, as shown by the expressions of cleaved-Caspase 3 and cleaved-PARP1 (Fig. 3g). Colony formation assays showed that HCC/SPR^D257G^ cells behaved similarly to the wild-type HCC cells in terms of proliferation (Fig. 3h). Together, SPR modulates HCC cell growth and apoptosis mainly via its nonenzymatic activity, instead of the catalytic activity.

### SPR controls Bim signaling in HCC cells

**Fig. 4:**
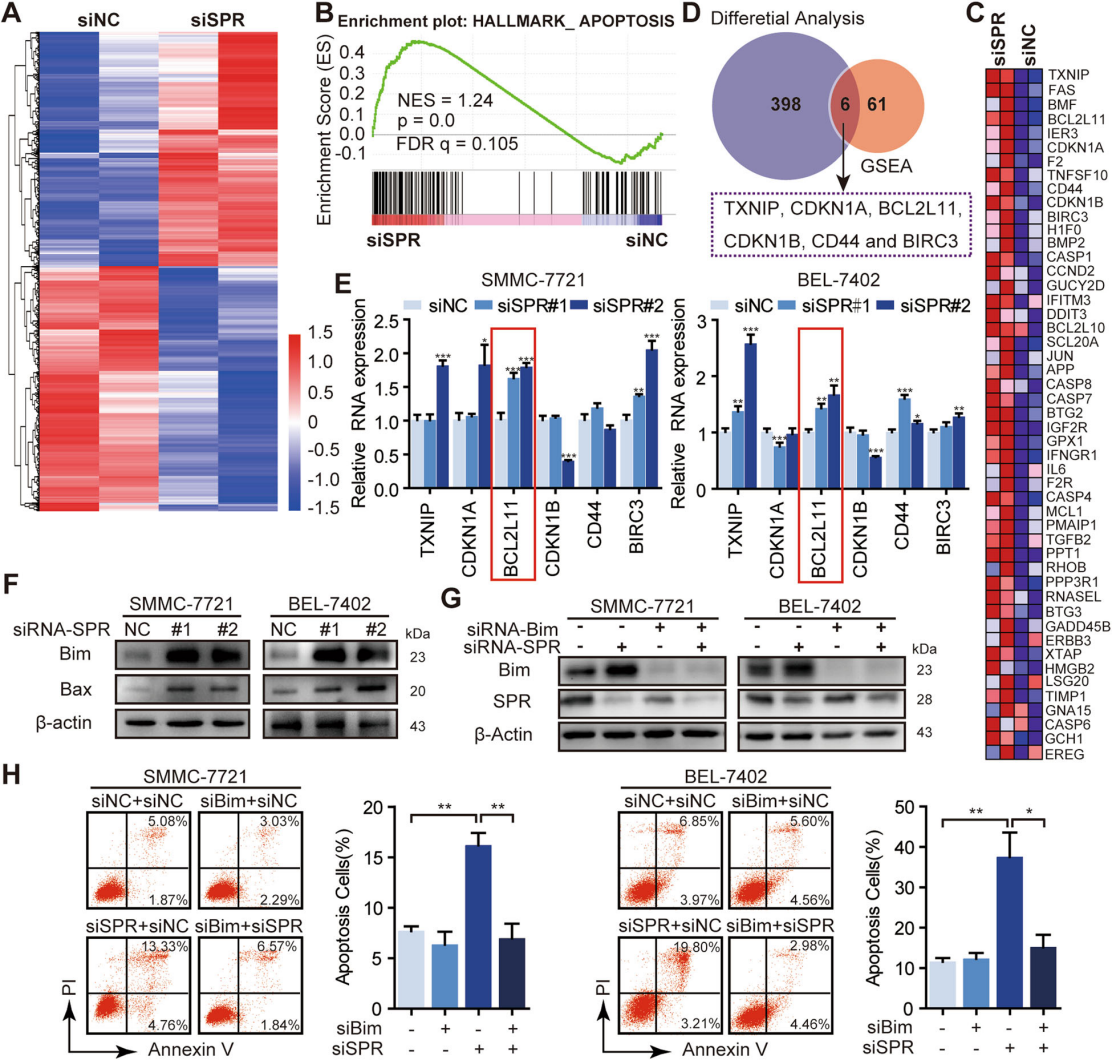
Bim was negatively regulated by SPR. **a** Heat map of the fold changes of gene expressions in SMMC-7721 cells induced by SPR knockdown detected by RNA-seq. **b** Biological process enrichment plots (using oncogenic “H” from the Molecular Signatures database) in RNA-seq data with siSPR treatment versus the negative control (siNC) treatment in SMMC-7721 cells. The plots indicate a significant (FDR *q* < 0.25) enrichment of apoptosis signatures after siSPR treatment. **c** GSEA analysis of RNA-seq data affected by SPR knockdown. A heat map showing the enriched genes in the apoptosis process from the Molecular Signatures database with siSPR compared to siNC-treated SMMC-7721 cells. **d** A Venn diagram showing the overlap of genes between differential analysis and GSEA analysis based on RNA-seq data. **e** The mRNA expression of *TXNIP*, *CDKN1A*, *BCL2L11*, *CDKN1B*, *CD44*, and *BIRC3* in HCC cells treated with siSPR or siNC by qPCR. **f** The protein levels of Bim and its downstream Bax in different groups were detected by western blots. **g** Bim expression was significantly inhibited after treatment with siBim. **h** Bim suppression abolished the induction of apoptosis in SPR-depleted HCC cells analyzed by flow cytometry using annexin V/PI staining. Data are represented as the mean ± SD of three independent experiments. The *p*-values < 0.05 were considered statistically significant for all tests.

To systematically examine the cellular pathways affected by SPR depletion, we performed RNA-seq of SMMC-7721 transfected with siSPR or control siRNA. A total of 223 genes were downregulated, while 181 genes were upregulated after SPR knockdown (Padj < 0.05; Fig. 4a). Conducting GSEA of the RNA-seq data, we found that the apoptosis-associated pathway was statistically enriched in the SPR-depleted HCC cells (FDR *q* < 0.25; Fig. 4b), and the top 50 related genes were also included (Fig. 4c). Additionally, six apoptosis-related genes, which were significantly altered after SPR depletion, were selected by comparing the results from differential analysis and GSEA of the RNA-seq data (Fig. 4d). These six genes, namely *TXNIP*, *CDKN1A*, *BCL2L11*, *CDKN1B*, *CD44*, and *BIRC3*, were further confirmed by qPCR (Fig. 4e). Among these genes, only *BCL2L11* (Bim) showed a significantly consistent increase trend in both SMMC-7721 and BEL-7402 cells. Consistently, we detected an approximately five-fold increase in Bim protein levels and its downstream effector, Bax, upon SPR knockdown (Fig. 4f), which suggested that the Bim expression level could be regulated by SPR in HCC cells.

In contrast to HCC cells, HCC/SPR^D257G^ cells showed no significant difference in Bim expression (Fig. S3A). Besides, the Bim expression level was not affected by SPR enzymatic inhibitors (Fig. S3B). Therefore, SPR-regulated Bim signaling was independent of its enzymatic activity. We next characterized the role of Bim in SPR-regulated apoptosis. Bim was knocked down in HCC cells simultaneously treated with siSPR (Fig. 4g). Moreover, Annexin V/PI analysis indicated that Bim silencing significantly reduced apoptosis that was triggered by SPR knockdown (Fig. 4h). Collectively, these data support the hypothesis that SPR affects HCC cell apoptosis by regulating Bim signaling, independently of its enzymatic activity.

### FoxO3a nuclear translocation is responsible for Bim expression induced by SPR depletion

**Fig. 5:**
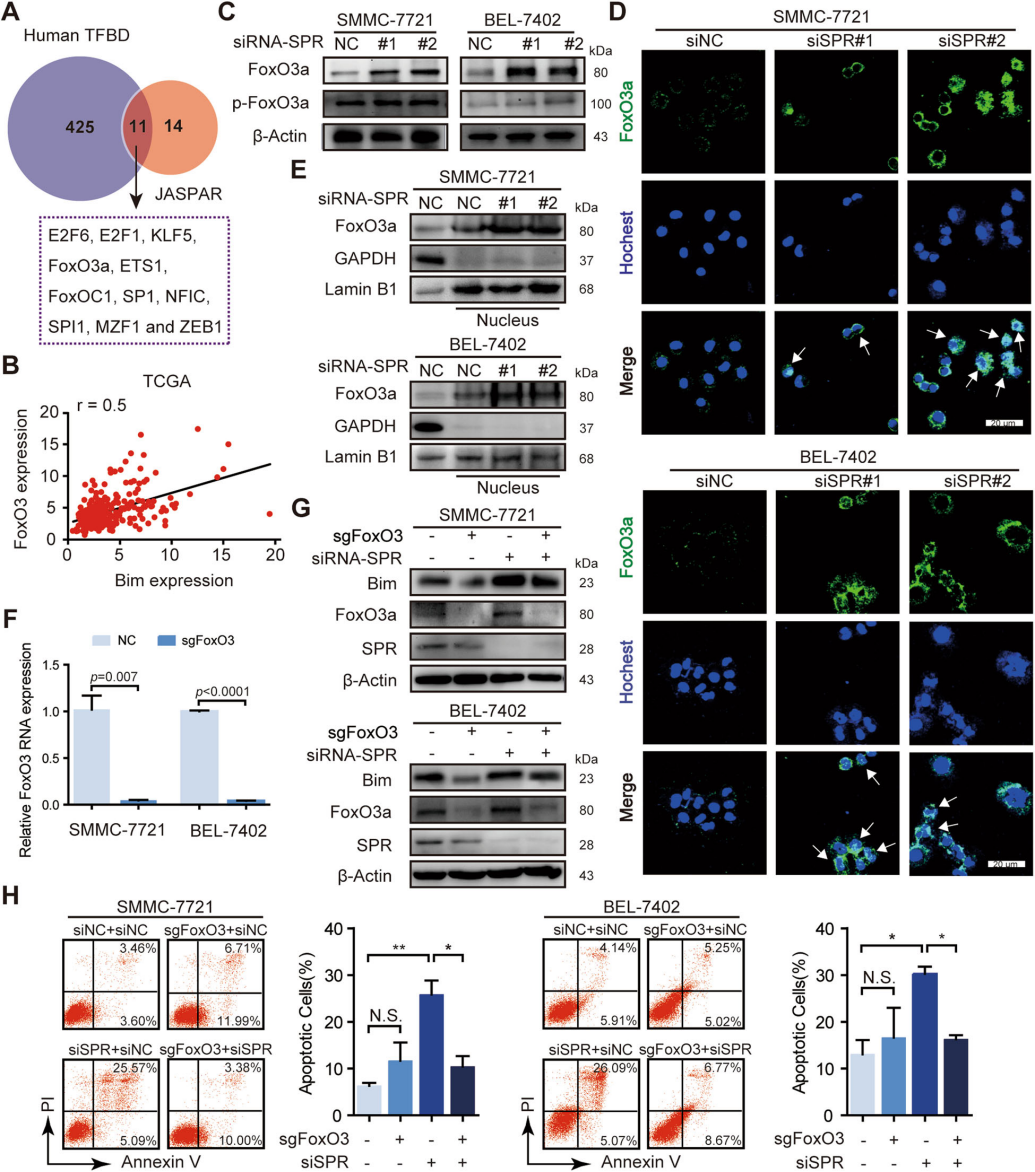
SPR regulates Bim expression via the nuclear translocation of FoxO3a. **a** Two bioinformatic prediction tools, humanTFDB and JASPAR, were used to find the transcription factors that could modulate Bim expression. **b** Bim mRNA expression in HCC tissues was significantly correlated with FoxO3a in TCGA datasets. **c** The expression levels of FoxO3a and p-FoxO3a were analyzed by western blots. SPR knockdown promoted the nuclear translocation of FoxO3a in HCC cells by separation of the cytoplasm and nucleus, as revealed by the **d** immunofluorescence assay and **e** western blots. Scale bar: 20 μm. **f** The knockout efficiency of CRISPR/Cas9 for FoxO3a was determined by qPCR. **g** FoxO3a suppression abolished the upregulation of Bim expression in SPR-depleted HCC cells. **h** Gene silencing of FoxO3a rescued the apoptosis induced by SPR depletion in HCC cells. Data are represented as the mean ± SD of three independent experiments. The *p*-values < 0.05 were considered statistically significant for all tests. NC negative control.

Given the previous results showed that SPR depletion activated Bim expression, we aim to identify the underlying mechanism by which SPR affected HCC cell growth and apoptosis via regulating Bim signaling. We first analyzed Bim phosphorylation, which has been confirmed to trigger Bim ubiquitination^30^. However, the levels of p-Bim and p-Erk were the same as in HCC cells treated with siSPR or control siRNA, suggesting that the increased Bim level in SPR-depleted HCC cells was not the result of phosphorylation (Fig. S3C). Because Bim levels can be controlled by transcriptional regulation, in addition to posttranslational modification, we investigated whether the upregulated Bim level in SPR-depleted HCC cells was the result of transcriptional regulation. Two bioinformatic tools, JASPAR (http://jaspar.genereg.net/) and humanTFDB (http://bioinfo.life.hust.edu.cn/HumanTFDB#!/), were used to identify the transcription factors that could modulate Bim expression. Superimposing the results obtained by the two algorithms, identified 11 transcription factors that could bind to the Bim promoter (Fig. 5a). In addition, TCGA data showed that FoxO3a and SP1 were positively associated with Bim mRNA levels (*r* ≥ 0.5; Figs. 5b; S3D). However, the protein levels of SP1 were unchanged after SPR depletion (Fig. S3E). Thus, FoxO3a might be a transcription factor for Bim expression regulated by SPR.

Precious studies have indicated that FoxO3a binds to the *Bim* gene and acts as a transcription promotor in HCC cells^31–33^. Therefore, we investigated the possible relationship between FoxO3a and SPR. Knockdown of SPR resulted in increased FoxO3a expression, but had no effect on p-FoxO3a level which obtains impaired capability of nuclear translocation (Fig. 5c). Otherwise, there was no consistent trend in the changes of the *FoxO3a* gene in different cells (Fig. S3F). Immunofluorescence staining was then employed to examine the subcellular localization of FoxO3a. The results showed that FoxO3a exhibited nuclear accumulation in SPR-depleted cells, but cytosolic localization in control cells (Fig. 5d). Western blot results consistently showed that SPR knockdown resulted in the prominent redistribution of FoxO3a to the nucleus (Fig. 5e). To further confirm the role of FoxO3a in upregulated Bim levels controlled by SPR, FoxO3a was knocked down in HCC cells transfected with siSPR (Fig. 5f). Increased Bim levels induced by SPR knockdown were substantially restored by depletion of FoxO3a protein (Fig. 5g). Importantly, the Annexin V/PI assay showed that FoxO3a silencing significantly reduced apoptosis that was initiated by SPR knockdown (Fig. 5h). Furthermore, deficiency of SPR enzymatic activity did not affect FoxO3a levels in HCC cells (Fig. S3A). In summary, a nonenzymatic function of SPR promotes Bim expression via inducing nuclear translocation of the FoxO3a protein.

### SPR regulates the growth of HCC xenografts

**Fig. 6:**
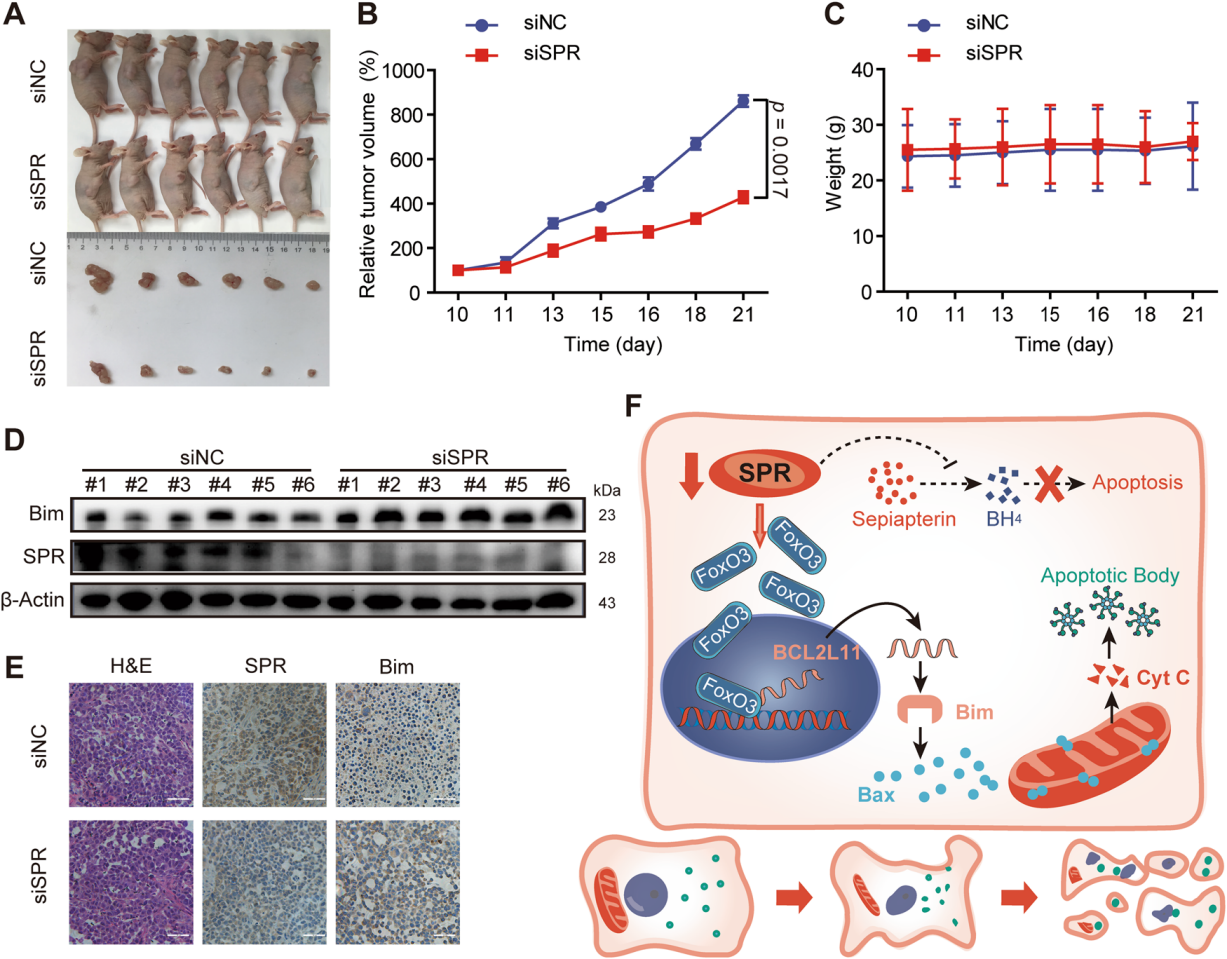
SPR depletion inhibits HCC growth in vivo. **a** The effects of SPR depletion on the tumor volumes of xenografts in nude mice. **b** Loss of SPR reduced the tumor growth rate. SMMC-7721 cells (1 × 10^6^ cells per mouse) were subcutaneously inoculated into the right flank of mice. When the tumor volume reached 100 mm^3^, siRNA (10 μg per tumor) was injected into tumors once every other day. **c** SPR knockdown had no effect on the body weight of mice. **d**, **e** The levels of SPR and Bim in different groups were detected by western blots and immunohistochemistry. Scale bar: 50 μm. **f** Schematic representation of the underlying mechanism of SPR based on this study. SPR regulates apoptosis of HCC cells via the FoxO3a/Bim-signaling pathway independently of its enzymatic activity. Each point represents the mean ± SD (*n* = 6). The *p*-values < 0.05 were considered statistically significant.

Above data highlighted the nonenzymatic function of SPR in modulating HCC cell proliferation and apoptosis, which is a pivotal determinant of cancer development. We further determined the role of SPR in HCC progression using a HCC xenograft model used in previous reports^34,35^. SMMC-7721 cells were subcutaneously xenografted into nude mice. After the tumor volume reached ~100 mm^3^, siSPR was injected into the liver cancer xenografts. Injection of siSPR reduced the tumor volume (Fig. 6a) and tumor growth rate (Fig. 6b), but resulted in no significant change in body weight (Fig. 6c). Furthermore, SPR knockdown in tumors was associated with increased levels of Bim (Fig. 6d,e). These data supported the conclusion confirmed by the in vitro assays, which showed that SPR played a critical role in regulating HCC proliferation. Collectively, our experiments unfold the underlying mechanism by which SPR regulates HCC cell apoptosis (Fig. 6f), and identify SPR as an important promoter for HCC development, which can facilitate better HCC diagnosis and treatments.

## Discussion

Although biological functions of SPR in BH_4_ biosynthesis have been well studied^11^, the potential roles and molecular mechanisms in cancer are still not widely recognized, especially in some high-incidence tumors. Meanwhile, the inability to suppress breast cancer cell growth^19^ and the potential side effects of SPR enzymatic inhibitors^21,22^ make it urgent to determine novel functions of SPR in tumor development. In this study, we investigated the potential biological functions of SPR in HCC progression. According to the results, high SPR expression was significantly associated with shorter survival of HCC patients, indicating that SPR could act as a potential prognostic factor of HCC. Moreover, we identified the crucial role of SPR in HCC cell growth and apoptosis by both cell biology experiments and a nude mice xenograft model. Importantly, the CRISPR/cas9 system was used to confirm that SPR modulated HCC cell growth and apoptosis via its nonenzymatic activity. Increasing evidence has shown that recognition of metabolically independent activities of enzymes provides a new way to reevaluate their roles in cancer progression^23–26^. Thus, there is a need to identify the nonenzymatic function of SPR. Our results indicated that SPR depletion promoted HCC apoptosis by activating the FoxO3a/Bim signaling pathway, suggesting that SPR could be used in a therapeutic strategy for HCC.

Previous reports have illustrated that SPR functions as regulator in NB^12–14^. However, the role of SPR in HCC has not been reported as far as we know. Here, we found that SPR was upregulated in HCC tissues (Fig. 1d). Furthermore, survival analyses showed that SPR overexpression predicted unfavorable overall survival in HCC patients (Fig. 1e). These results suggest that SPR could be a potential clinical prognostic indicator of HCC. Moreover, in vitro assays such as real-time cell analysis (Fig. 2e), and in vivo functional experiments (Fig. 6b) indicated that SPR depletion significantly inhibited HCC proliferation and promoted cancer cell apoptosis through the mitochondrial pathway. Therefore, we provide evidence showing the critical role of SPR as a key contributor to the progression of HCC.

Because the catalytic activity of SPR is pivotal for maintaining the normal physiological function of the nervous system by regulating BH_4_ biosynthesis^21,36^, we first investigated the role of SPR enzymatic activity in the proliferation and apoptosis of HCC cells. Strikingly, the results demonstrated that suppression of SPR catalytic activity by inhibitors (Fig. 3b, c) or infection with a gene-edited system (Fig. 3g, h) had no effect on cancer cell proliferation and apoptosis; previous data from MOLT-4 and MCF-7 cancer cells treated with an SPR inhibitor partially supported our findings^19^. Consistently, the addition of BH_4_ did not restore the apoptosis induced by SPR knockdown (Fig. 3d). All these results suggest that, in HCC cells, SPR regulates cell proliferation and apoptosis in a nonenzymatic manner. However, sulfasalazine, an SPR enzymatic inhibitor, was reported to suppress the growth of NB cells in vitro and in vivo^12^. The reason for the different functions of SPR enzymatic activity in NB and HCC cells might be that SPR enzymatic activity was bypassed by aldose reductase and carbonyl reductase in the liver and other peripheral tissues, rather than in the central nervous system^20^. In brief, this study extends our understanding of SPR by showing that SPR nonenzymatically regulates HCC proliferation and apoptosis.

Enzymatically independent activities of enzymes, such as PGAM-1 and LSD1, provide potential targets for clinical tumor therapy^23–26^. However, the mechanism of SPR’s nonenzymatic function remains largely unknown. Therefore, we expected to identify the molecular mechanism of SPR’s nonenzymatic function in HCC. According to RNA-seq data, apoptosis pathways were dramatically affected by SPR knockdown (Fig. 4a, b). Among them, the level of Bim, which can initiate the intrinsic apoptotic pathway under both physiological and pathophysiological conditions^37^, as well as its downstream Bax, were upregulated upon SPR depletion (Fig. 4f). Moreover, SPR enzymatic inhibition did not affect Bim expression. Collectively, SPR exerted an apoptosis-promoting role in HCC by activating the expression of Bim. Notably, the nonenzymatic activity of SPR is likely not limited to that observed in this study. The SPR-associated gene identified by RNA-seq, although needing further validation, suggests its broad involvement in different cellular processes, such as migration.

Numerous reports have suggested that phosphorylation is an important factor in the posttranslational regulation of Bim^38^; therefore, we analyzed the levels of p-Bim. However, no difference was found in p-Bim expression. Next, we investigated the transcriptional regulation of Bim. There was a significant association between Bim levels and the expression of SP1 or FoxO3a in HCC, based on TCGA dataset (Figs. 5b; S3D). Otherwise, SPR knockdown did not affect SP1 protein levels (Fig. S3E). Additionally, it has been reported that FoxO3a plays a critical role in controlling Bim expression in HCC cells^32,33^. Therefore, we measured the levels of FoxO3a in HCC cells, and found that FoxO3a expression was upregulated by SPR depletion (Fig. 5c). Moreover, immunofluorescence analysis demonstrated that SPR knockdown promoted the nuclear translocation of FoxO3a (Fig. 5d). Collectively, our results suggested that SPR knockdown in HCC cells promoted apoptosis via the FoxO3a/Bim-signaling pathway. The characterization of SPR’s nonenzymatic functions in HCC could provide a possible therapeutic strategy for HCC; therefore, nonenzymatic sites and inhibitors need to be investigated in future studies.

In the present study, we explored the involvement of SPR’s nonenzymatic activity in cell apoptosis. According to the data from RNA-seq, the nonenzymatic role of SPR in additional biological events needs to be further explored. Moreover, because of the significant role of SPR’s nonenzymatic activity in cancer progression, nonenzymatic sites and inhibitors should also be further investigated. Importantly, this study identified an important role of SPR in HCC and provided a potential clinical prognostic indicator for HCC. In addition, we featured the nonenzymatic role of SPR in HCC progression. The determination of the enzymatically independent functions of SPR supports the development of drugs that have fewer side effects caused by the inhibition of enzymatic activity^21,22^. Furthermore, the underlying signaling pathway of SPR’s nonenzymatic activity improves our understanding of SPR and provides a possible therapeutic strategy for HCC.

## Supplementary information

Supplementary Figure 3

Supplementary Figure Legends

Supplementary Figure 1

Supplementary Figure 2
