# Supplementary figures and images for: Sepiapterin reductase promotes hepatocellular carcinoma progression via FoxO3a/Bim signaling in a nonenzymatic manner
Source: Cell Death Dis. 2020 Apr 20;11(4):248. doi: 10.1038/s41419-020-2471-7 (PMC7170898; doi:10.1038/s41419-020-2471-7)

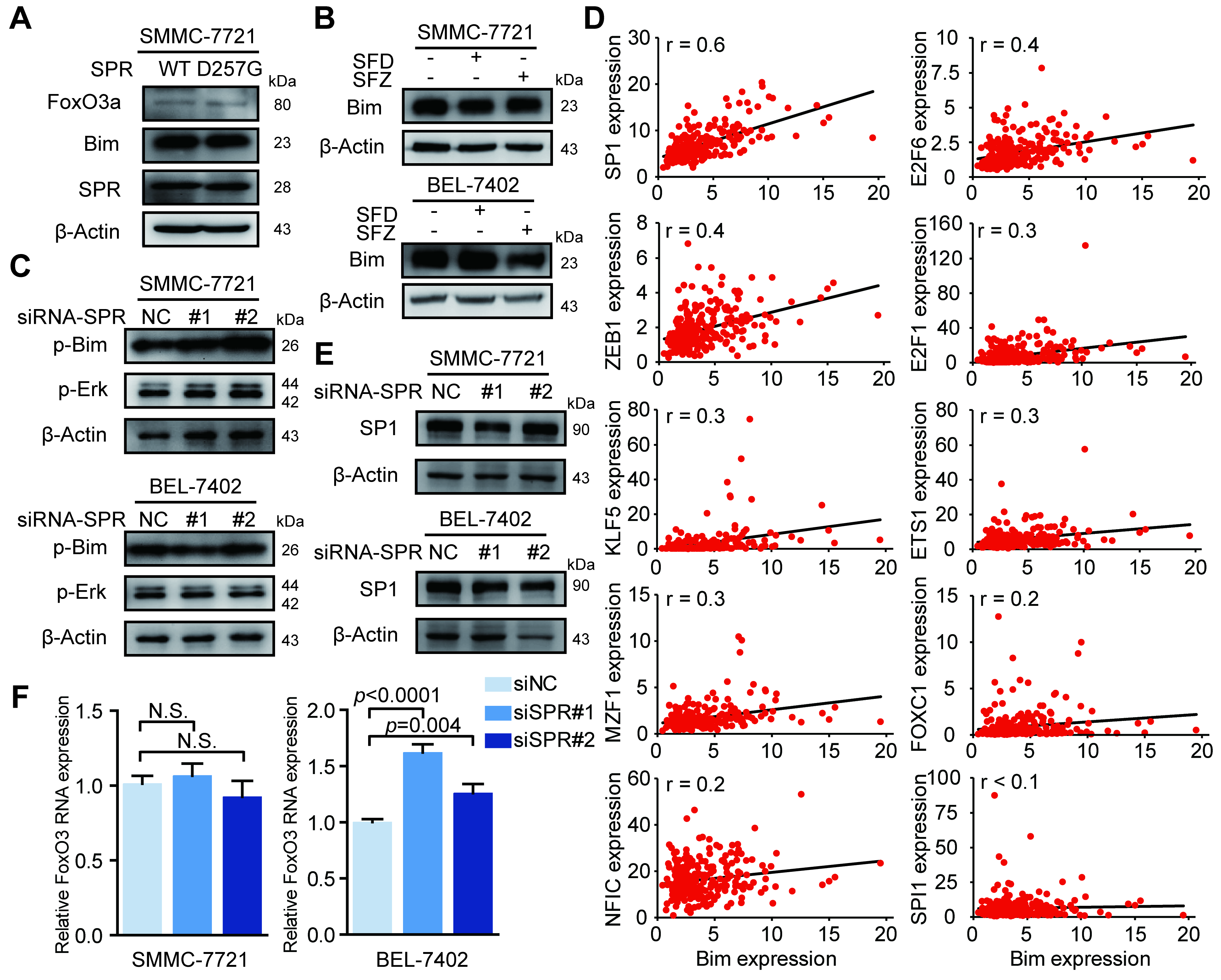

Supplement: Supplementary file 1 — Supplementary Figure 3 [file 41419_2020_2471_MOESM1_ESM.tif]

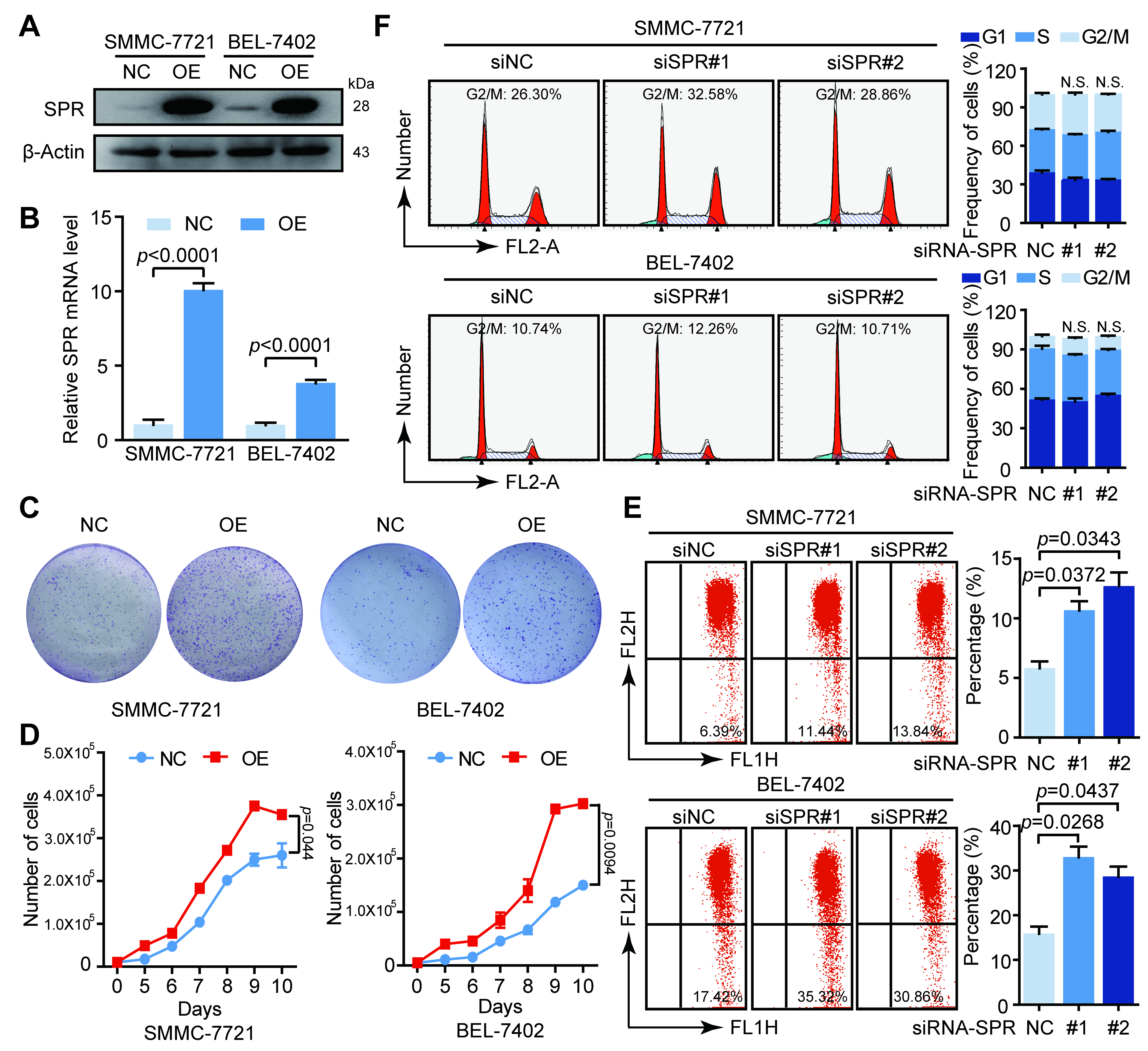

Supplement: Supplementary file 3 — Supplementary Figure 1 [file 41419_2020_2471_MOESM3_ESM.tif]

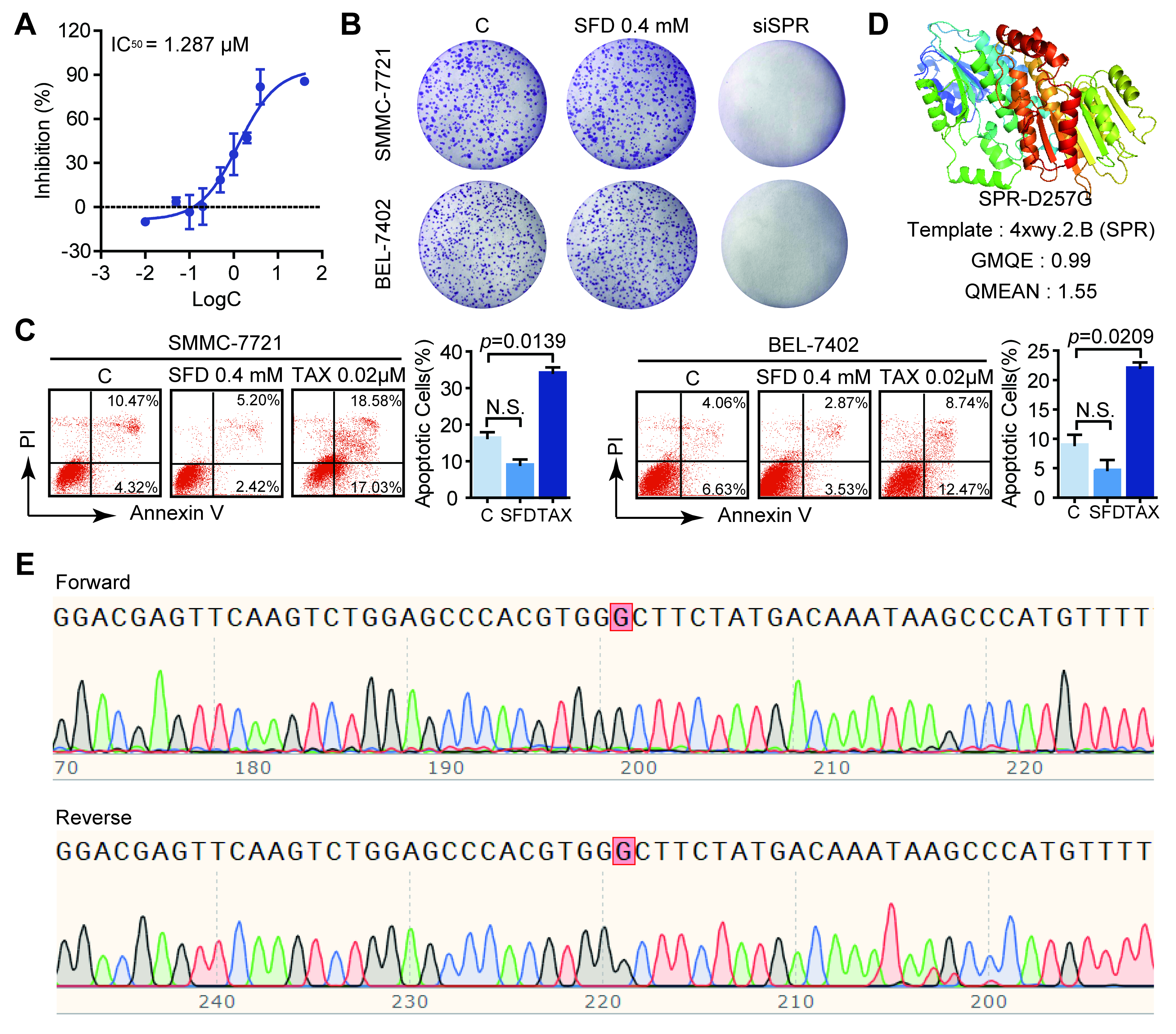

Supplement: Supplementary file 4 — Supplementary Figure 2 [file 41419_2020_2471_MOESM4_ESM.tif]
