# Supplementary material for: Sepiapterin reductase promotes hepatocellular carcinoma progression via FoxO3a/Bim signaling in a nonenzymatic manner
Source: Cell Death Dis. 2020 Apr 20;11(4):248. doi: 10.1038/s41419-020-2471-7 (PMC7170898; doi:10.1038/s41419-020-2471-7)
Supplement: Supplementary file 2 — Supplementary Figure Legends [file 41419_2020_2471_MOESM2_ESM.docx]

**Figure S1. (A-B)** Overexpression of SPR (OE) in transfected HCC cell lines verified by western blots and qPCR compared to that of cells transfected with the control vector (NC). **(C-D)** The effect of SPR overexpression on HCC cells proliferation was analyzed by colony formation and proliferation curve assays. **(E)** Mitochondrial membrane potential in HCC cells was detected by flow cytometry with JC-1. **(F)** Cell cycle distribution was detected after SPR knockdown. Data are represented as the mean ± S.D. of three independent experiments. The *p*-values < 0.05 were considered statistically significant for all tests.

**Figure S2. (A)** The inhibitory activities of sulfapyridine (SFD) against human SPR were measured in a spectrophotometric assay. **(B)** Effects of sulfapyridine on cell viability of SMMC-7721 and BEL-7402 cells were detected by colony formation assay. **(C)** Cell apoptosis was detected by flow cytometry after treated with sulfapyridine at indicated concentrations for 96 hours in SMMC-7721 and BEL-7402 cells. **(D)** The structure of indicated SPR mutant was predicted by homology modeling. **(E)** Sequence results (forward and reverse) of SMMC-7721/SPR D257G. Data are represented as the mean ± S.D. of three independent experiments. The *p*-values < 0.05 were considered statistically significant for all tests. TAX: paclitaxel.

**Figure S3. (A)** The protein levels of SPR, Bim and FoxO3a in SMMC-7721/SPR D257G were compared to these in SMMC-7721 by western blots. **(B)** The inhibitors of SPR enzymatic activity, including sulfathiazole (SFZ) and sulfapyridine (SFD), had no effect on the expression of Bim. **(C)** The protein levels of p-Bim and p-Erk were detected in HCC cell lines treated with siSPR or siNC by western blots. **(D)** The correlation between Bim mRNA and different transcription factors mRNA, containing SP1, E2F6, ZEB1, E2F1, KLF5, ETS1, MZF1, FOXC1, NFIC and SPI1, in HCC tissues was analyzed based on the TCGA datasets. **(E)** The levels of SP1 in different groups were detected by western blots. **(F)** FoxO3a gene expression was analyzed by qPCR in HCC cells transfected with siSPR or siNC. Data are represented as the mean ± S.D. of three independent experiments. The *p*-values < 0.05 were considered statistically significant for all tests. WT: wide type; NC: negative control.
